# Local Crop Planting Systems Enhance Insecticide-Mediated Displacement of Two Invasive Leafminer Fly

**DOI:** 10.1371/journal.pone.0092625

**Published:** 2014-03-20

**Authors:** Yulin Gao, Stuart R. Reitz, Qingbo Wei, Wenyan Yu, Zhi Zhang, Zhongren Lei

**Affiliations:** 1 State Key Laboratory for Biology of Plant Diseases and Insect Pests, Institute of Plant Protection, Chinese Academy of Agricultural Sciences, Beijing, P. R. China; 2 Department of Crop and Soil Sciences, Malheur County Extension, Oregon State University, Ontario, Oregon, United States of America; Federal University of Viçosa, Brazil

## Abstract

*Liriomyza sativae* and *L. trifolii* are highly invasive leafminer pests of vegetable crops that have invaded southern China in recent years. *Liriomyza sativae* was the first of these species to invade China, but it is now being displaced by *L. trifolii*. The rate and extent of this displacement vary across southern China. In Hainan, monocultures of highly valuable cowpea are planted and treated extensively with insecticides in attempts to control leafminer damage. In Guangdong, cowpea fields are interspersed with other less valuable crops, such as towel gourd (*Luffa cylindrica*), which receive significantly fewer insecticide applications than cowpea. To determine how differences in cropping systems influence the *Liriomyza* species composition, we conducted field trials in 2011 and 2012 in Guangdong where both species were present. We replicated conditions in Hainan by planting cowpea monocultures that were isolated from other agricultural fields, and we replicated conditions in Guangdong by planting cowpea in a mixed crop environment with towel gourd planted in neighboring plots. We then compared leafminer populations in cowpea treated with the insecticide avermectin and untreated cowpea. We also monitored leafminer populations in the untreated towel gourd. Untreated cowpea and towel gourd had comparatively low proportions of *L. trifolii*, which remained relatively stable over the course of each season. Avermectin applications led to increases in the proportions of *L. trifolii*, and after three weekly applications populations were >95% *L. trifolii* in both crop systems. However, the rate of change and persistence of *L. trifolii* in the mixed crop system were less than in the monocrop. These results indicate that *L. trifolii* is much less susceptible to avermectin than is *L. sativae*. Further, *L. sativae* was able to persist in the untreated towel gourd, which probably enabled it to recolonize treated cowpea.

## Introduction

Leafmining flies in the genus *Liriomyza* Mik (Diptera: Agromyzidae) are among the most important invasive pests of vegetable crops throughout the world. Damage occurs primarily as larvae feed within host plant foliage [1]. The most widespread and successful invasive *Liriomyza* species are *L. sativae* Blanchard, *L. trifolii* (Burgess) and *L. huidobrensis* (Blanchard) [2], and these have recently emerged as particularly damaging pests in China, where they have had a complex history of invasion and interspecific interactions [3], [4]. *Liriomyza sativae* was found first in Hainan Province in October 1993, and then spread throughout China within a few years [5]. By 2005, the distribution of *L. sativae* encompassed more than 30 provinces, and it has since been regarded as an established exotic species in China. *Liriomyza trifolii* is a more recent invasive that was first recorded in Guangdong and Hainan Provinces in 2005 and 2006, respectively [6].

After their respective invasions, both of these highly polyphagous species became the most important pests of cowpea (*Vigna uniguilata*), towel gourd (*Luffa cylindrica*), cucumber (*Cucumis sativus*), and many other vegetable crops in southern China. However since its establishment, *L. trifolii* has begun to displace *L. sativae* in certain areas across southern China [3], [7]. Gao et al. [7] proposed that *L. trifolii* in southern China are less susceptible to insecticides, specifically avermectin and cyromazine, than is *L. sativae*, and this difference in susceptibility is a major factor in the displacement of *L. sativae* by *L. trifolii*.

Although the displacement of *L. sativae* by *L. trifolii* has been rapid in certain areas of southern China, such as on Hainan Island, in other regions the displacement has not been as rapid or drastic. Displacements of one species by another are likely the outcome of interactions among multiple mechanisms [8]. Therefore, geographic differences in the demographic composition of *Liriomyza* populations could be a result of differences in cropping systems and interactions between cropping systems and insecticide use patterns.

In southern China, mixed plantings of cowpea, towel gourd, and cucumber are common, but the relative abundance of cowpea to non-cowpea crops is highly variable across regions. For example, the period from December to April corresponds to the time of when *Liriomyza* populations peak in Hainan Province, and during this time, cowpea is intensively planted so that it comprises 70–90% of the total acreage of *Liriomyza* susceptible crops (i.e. the proportion of cowpea, calculated by dividing the acreage of cowpea by the total acreage of the main host crops of *Liriomyza* in a region). Because cowpea is the most economically valuable vegetable crop in Hainan, farmers attempt to control leafminer populations through intensive insecticide use. Grower surveys have shown that avermectin and cyromazine account for >95% of the total insecticides used against leafminers [3]. Of these two, avermectins are the most commonly used insecticides in southern China.

In contrast to the predominance of cowpea in Hainan, in Guangdong province, mixed plantings of cowpea, towel gourd, cucumber and other *Liriomyza*-susceptible crops are common during the peak time for *Liriomyza* populations there, which is from April to June. In particular, mixed plantings of cowpea and towel gourd are a typical cropping system in Guangdong. On average, cowpea comprises about 15% of the acreage of *Liriomyza* hosts in Guangdong, and towel gourd comprises about 40% of the acreage. Also in contrast to Hainan Province, overall insecticides use is not as intensive in Guangdong Province [9]. With mixed cropping systems, farmers do not treat all crops with the same amount and frequency of insecticides. Less valuable crops, such as towel gourd, tend to be treated less than the high-value cowpeas [10]. Therefore, some non-cowpea host crops, such as towel gourd, may serve as refuge hosts for *Liriomyza sativae* when cowpea is intensively treated with insecticides.

To characterize how insecticide use and cropping systems interact to affect the demographic composition of the *Liriomyza* species complex in southern China, we conducted a series of surveys to monitor species composition across southern China and field experiments in different cropping systems. We were specifically interested in testing the hypotheses that use of the insecticide avermectin would increase the proportion of *L. trifolii* in populations, but that the effect of the insecticide would be affected by cropping system.

## Results

### Field surveys in Guangdong and Hainan Provinces

In the 2011 survey, we collected 28 samples of leafminers (>30 individuals per sample) from cowpea crops in the eight locations of Guangdong Province. Almost all samples contained both species of leafminers. Overall, *L. sativae* comprised 58% of the total *Liriomyza* population (n = 1055), with the remaining 42% all being *L. trifolii*. Although these results show clearly that both *L. trifolii* and *L. sativae* were prevalent in Guangdong Province, *L. sativae* made up a significantly greater proportion of the *Liriomyza* complex than did *L. trifolii* (*x* ^2^ = 27.1, df = 1, P<0.0001).

From January through March of 2011, we collected 24 samples of leafminers (>30 individuals per sample) from cowpea crops in eight locations of Hainan Province. The survey results indicated that almost all of the leafminers (n = 804) were *L. trifolii* (99.8%). No *L. sativae* individuals were found, and only two *L. huidobrensis* individuals were found. These results show clearly that *L. trifolii* has replaced *L. sativae* as the dominant *Liriomyza* species on this cowpea-intensively planting system (*x* ^2^ = 798.0, df  = 1, P<0.0001).

### Field population experiments in 2011

**Figure 1:**
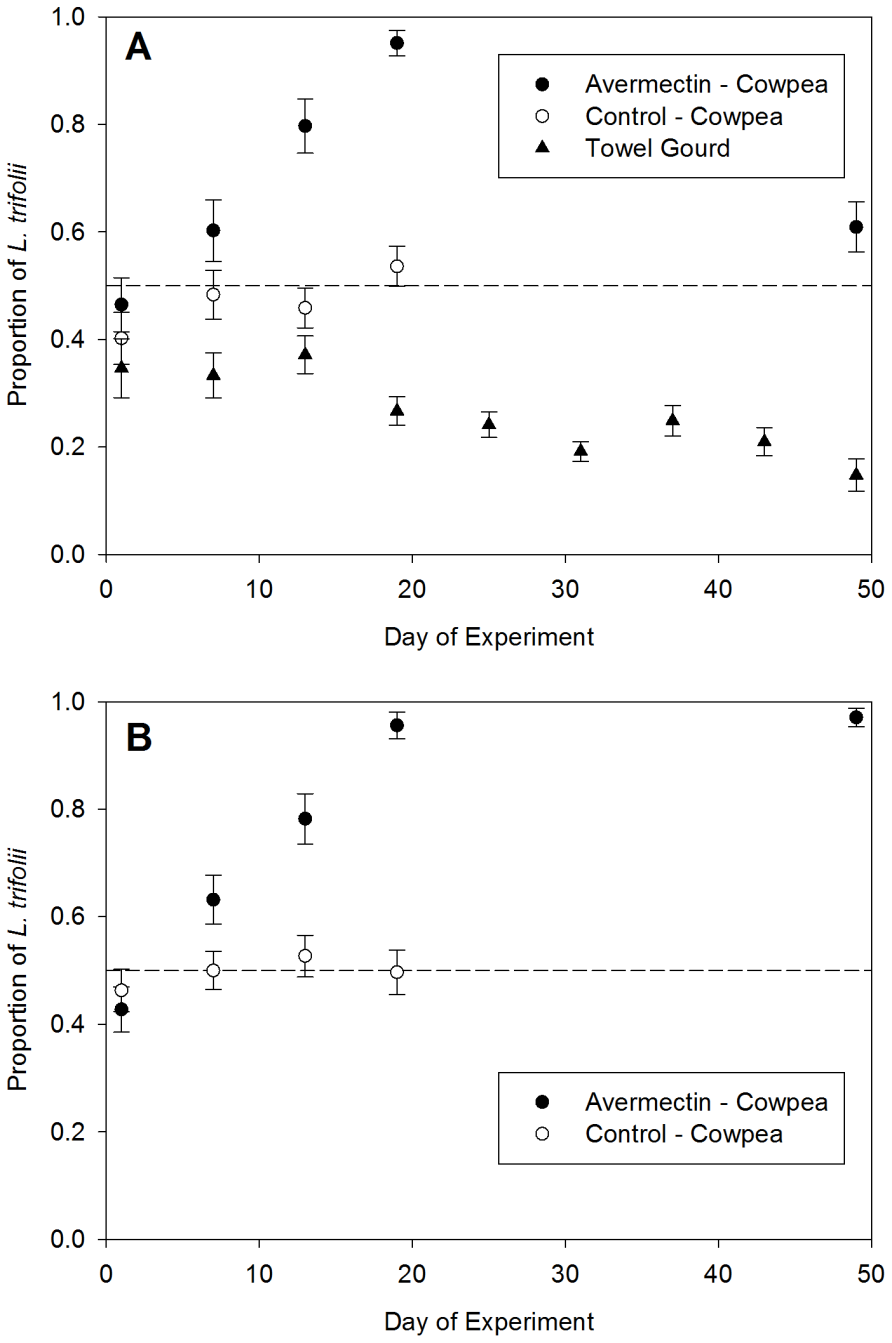
*Liriomyza* species composition during 2011. (A) mixed cropping systems of avermectin-treated and untreated cowpea (*Vigna uniguilata*), and adjoining plantings of towel gourd (*Luffa cylindrica*), and (B) plantings of cowpea that were treated with avermectin or were untreated controls. Avermectin treatments were made on days 1, 7, and 13 of the experiment following *Liriomyza* sample collections. Data points represent means ± SEM.

Field trials were conducted in a region of Guangdong Province where both *L. sativae* and *L. trifolii* were present. In 2011 trials, the use of avermectin led to significant increases in the proportion of *L. trifolii* in cowpea (Figure 1A and 1B). In the mixed plantings of cowpea and towel gourd, there was a significant treatment x time interaction demonstrating that the composition of populations changed with continued insecticide applications (F = 7.80 df  = 3, 16, P = 0.002). On the sample date before insecticide treatments began (day 1 of the experiment), the mean percentage of *L. trifolii* in the cowpea plots to be treated with avermectin was 46.5±5.0%, with all other leafminers being *L. sativae*. In the sample collections following the use of avermectin (days 7, 13, 19 of the experiment) the proportion of *L. trifolii* increased to 95.1±2.4% on day 19 (test for linear increase: t = 6.06, df  = 16, P<0.0001). In the cowpea plots not treated with avermectin, the initial percentage of *L. trifolii* was 40.2±4.9%, with all other leafminers being *L. sativae*. This percentage was not significantly different from that in the avermectin treated plots on day 1 (P = 0.38). However, there was no significant change in the proportion of *L. trifolii* over time in the untreated cowpea plots (t = 1.93, P = 0.07). The proportion of *L. trifolii* in the towel gourd plots actually declined significantly over time, going from 34.7±5.5% of the population at day 1 to 14.8±3.0% at day 49 of the experiment (F = 5.20, df  = 8, 18, P = 0.0018).

In the cowpea monoculture plantings, there also was a significant treatment x time interaction (F = 9.95, df = 3, 16, P<0.0001), indicating that the proportions of *L. trifolii* in populations were affected by insecticide treatments. On day 1 of this experiment (before insecticide applications began), there was no significant difference in the composition of the leafminer populations between the insecticide and untreated plots (P = 0.55). In the avermectin treatment, 42.8±4.2% of the leafminers were *L. trifolii*, and 46.3±3.9% of the leafminers were *L. trifolii* in the untreated plots. There was no significant change in the proportion of *L. trifolii* over time in the untreated plots (test for linear trend: t = 0.71, df  = 16, P = 0.49). In contrast, there was a significant linear increase in the proportion of *L. trifolii* over days 1–19 in the plots treated with avermectin (t = 5.78, df  = 16, P<0.0001).

The last avermectin application was made after samples were collected on day 13 of the experiments. In the mixed culture plantings, the proportion of *L. trifolii* in the avermectin treatment declined significantly from day 19 to day 49, when the population was only 60.9±4.7% *L. trifolii* (Figure 1A; t = 4.61, df  = 4, P = 0.01). However, in the cowpea monoculture, the proportion of *L. trifolii* remained at very high levels (97. 1±1.7%) after insecticide applications were discontinued (Figure 1B; comparison of proportions between day 19 and day 49: t = −0.51, df  = 4, P = 0.64).

### Field population experiments in 2012

The 2012 trials gave similar results to the 2011 trials; however, the initial proportions of *L. trifolii* were higher in 2012 than in 2011. The overall percentage of *L. trifolii* in cowpeas at the beginning of the 2012 experiments was 54.9±4.0% in the mixed plantings and 62.6±4.1% in the cowpea monoculture compared with 43.3±3.5% and 44.5±2.9%, respectively at the beginning of the 2011 experiments. The overall percentage of *L. trifolii* in towel gourd plots was 40.0±6.9% in 2012 compared with 34.7±5.5% in 2011.

**Figure 2:**
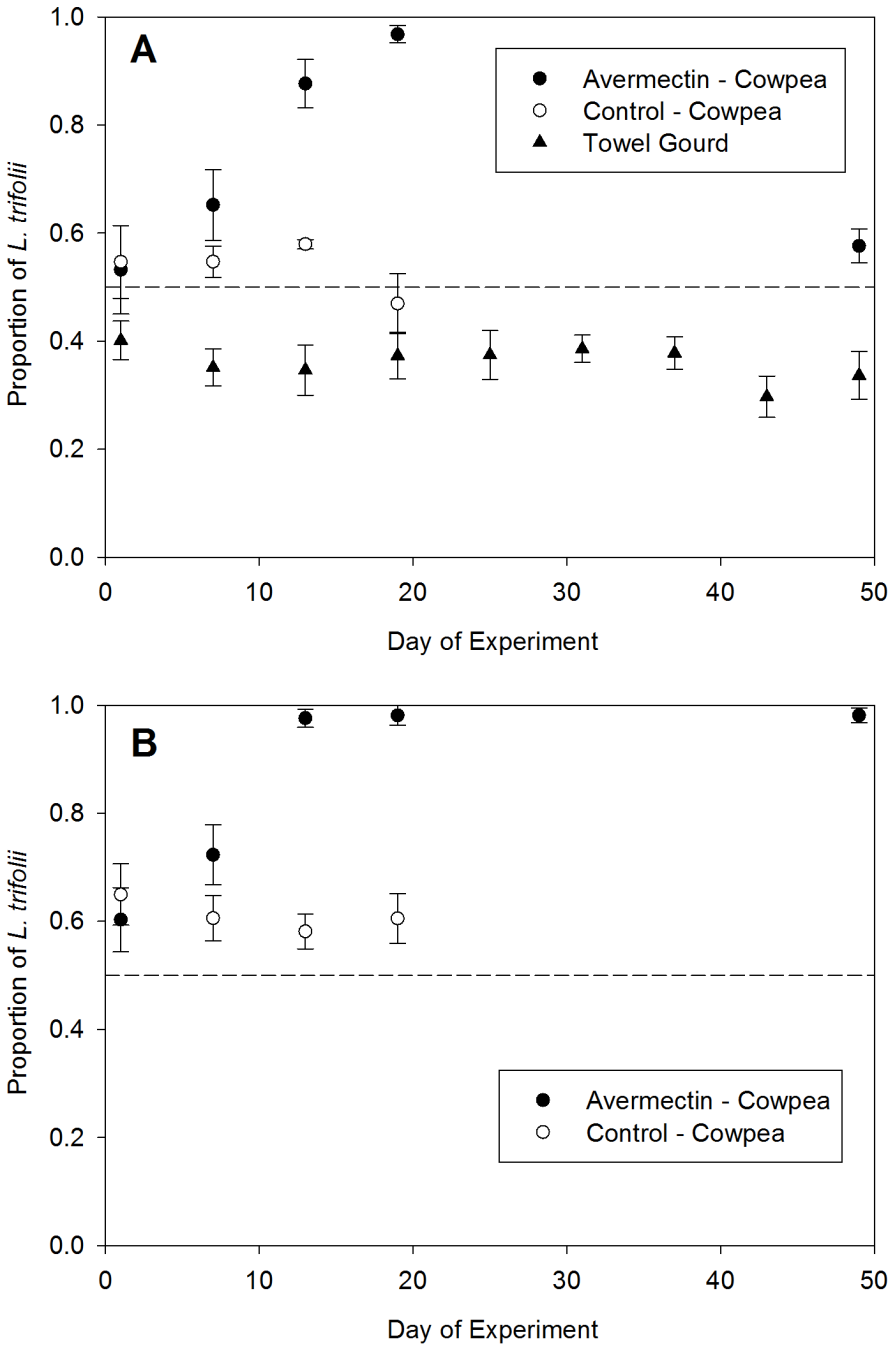
*Liriomyza* species composition during 2012. (A) mixed cropping systems of avermectin-treated and untreated cowpea, and adjoining plantings of towel gourd (*Luffa cylindrica*), and (B) plantings of cowpea that were treated with avermectin or were untreated controls. Avermectin treatments were made on days 1, 7, and 13 of the experiment following *Liriomyza* sample collections. Data points represent means ± SEM.

**Figure 3:**
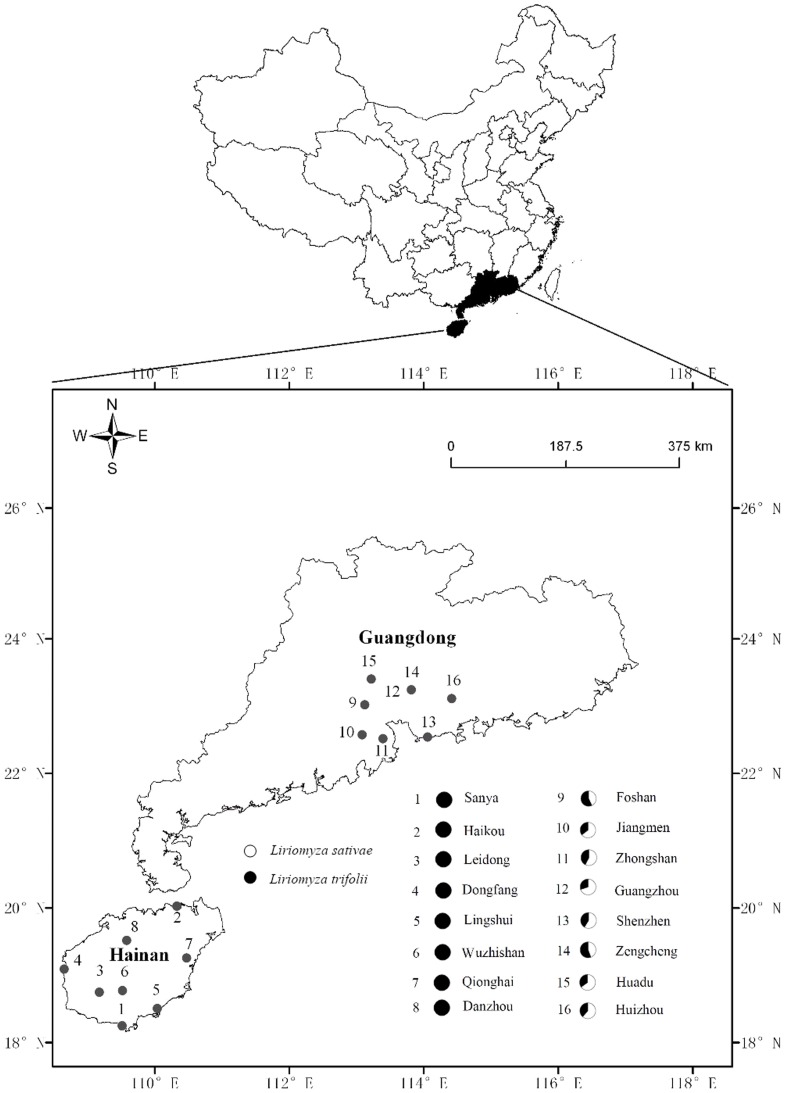
Map showing field sites in Hainan Province and Guangdong Province where cowpeas (*Vigna uniguilata*) were surveyed for *Liriomyza* species during 2011.

In 2012, the use of avermectin led to significant increases over time in the proportions of *L. trifolii* in cowpea (Figures 2A and 2B). In mixed plantings of cowpea and towel gourd, there was a significant treatment x time interaction demonstrating that the composition of populations changed with continued insecticide applications (F = 9.23, df  = 3, 16, P = 0.0009). There was a significant linear increase in the mean percentage of *L. trifolii* in the cowpea plots that were treated with avermectin, as the percentage in these plot went from 54.2±5.9% on day 1 to 96.6±2.4% on day 19 (test for linear increase: t = 4.74, df  = 16, P = 0.0002). In the untreated cowpea plots, the initial percentage of *L. trifolii* was 55.6±5.9%, with all other leafminers being *L. sativae*. This percentage was not significantly different from that in the avermectin treated plots on day 1 (P = 0.87). However, there was no significant change in the proportion of *L. trifolii* over time in the untreated cowpea plots (Figure 3; test for linear change in proportion of *L. trifolii*: t = −1.14, P = 0.27). The percentage of *L. trifolii* in the towel gourd plots ranged from 30–40% over the entire trial (F = 0.92, df  = 8, 18, P = 0.52).

In the cowpea monoculture plantings, there also was a significant treatment x time interaction (F = 9.24, df  = 3, 16, P = 0.0009), indicating that the proportions of *L. trifolii* in populations were affected by insecticide treatments, as occurred in the mixed crop experiment. On day 1 of this experiment (before insecticide applications began), there was no significant difference in the composition of the leafminer populations between the insecticide-treated and untreated plots (P = 0.59). The proportion of *L. trifolii* in the untreated plots remained relatively constant from day 1 to day 19, as there was no significant linear trend in this treatment (t = 0.67, df = 16, P = 0.51). In contrast, there was a significant linear increase in the proportion of *L. trifolii* over days 1–19 in the plots treated with avermectin (t = 4.15, df  = 16, P = 0.0007).

The last avermectin application was made after samples were collected on day 13 of the experiments. As occurred in 2011, in the mixed culture plantings, the proportion of *L. trifolii* in 2012 in the avermectin treatment declined significantly from day 19 to day 49, when the population was only 57.6±3.2% *L. trifolii* (Figure 2A; t = 4.16, df  = 4, P = 0.016). However, in the cowpea monoculture, the proportion of *L. trifolii* remained at very high levels (98. 1±1.3%) after insecticide applications were discontinued (Figure 3B; comparison of proportions between day 19 and day 49: t = 0.01, df  = 4, P = 0.99).

## Discussion

Our surveys showed that *L. trifolii* has become the predominant leafminer species within cowpea crops in Hainan Province and that *L. sativae* is rare, if present at all. Although *L. trifolii* has been established for a longer time in Guangdong Province than in Hainan, it has not been as successful in invading Guangdong and displacing *L. sativae. Liriomyza sativae* was still the predominant species in Guangdong where cropping systems are more diversified and complex, and insecticide use is less intensive than in Hainan. The results of our field experiments demonstrate that insecticide use can rapidly and dramatically alter the species composition of *L. sativae* and *L. trifolii*, and therefore, insecticide use patterns may be responsible for shifting the species balance in favor of *L. trifolii*. However, the continued predominance of *L. sativae* in Guangdong suggests that environmental differences allow it persist there.

In our field trials, we sought to replicate cropping environments similar to those in Hainan and Guangdong to assess how insecticide treatments and cropping systems may affect the demographics of these two invasive pest species. To this end, we established field sites with a cowpea monoculture, as is typically found in Hainan, and a more diversified cropping system that included cowpea and towel gourd, as is typically found in Guangdong. Conducting the studies in a location with significant abundance of both species allowed us to better characterize interspecific dynamics and more easily track demographic changes than at an extremely early or late stage in the process when one or the other species may be relatively rare.

In the absence of insecticide pressures, the two *Liriomyza* species coexisted over the two years of our study. In both years, there were greater proportions of *L. sativae* than *L. trifolii* in the towel gourd plantings. This result is not surprising as the species show distinct host plant affinities [1], [11], [12], and *L. sativae* often predominates over *L. trifolii* in cucurbit crops [13]. However, *L. trifolii* comprised a greater proportion of the population in the towel gourd plantings in 2012 (*x*±95% confidence interval: 36.1±2.5% for the season) than it did in 2011 (26.4±4.6%), suggesting that the overall population composition at the study sites had changed over time.

The most dramatic changes in species composition that we observed were results of avermectin treatments. The leafminer complex rapidly shifted from approximately equal proportions of the two species to overwhelming proportions of *L. trifolii* within three weeks of avermectin applications. However, in the mixed crop system, *L. sativae* began to recolonize the insecticide-treated cowpea plots within 30 days after insecticide treatments had ceased, but recolonization of insecticide-treated plots by *L. sativae* took longer in the monocrop cowpea system.

Recolonization by *L. sativae* was also less successful following the 2011 experiment. The proportions of *L. trifolii* in cowpea plantings were higher at the beginning of the 2012 season than at the beginning of the 2011 season in both the mixed crop and monocrop systems. The change from the beginning of the first season to the beginning of the second season was greater for the monocrop cowpea system than for the mixed cropping system. These results provide evidence that *L. trifolii* is less susceptible than *L. sativae* to avermectin, and that differential effects of insecticides can drive significant long term changes in populations. Palumbo et al. [14] also argued for differential susceptibility driving demographic shifts in leafminers. They observed that *L. sativae* was the most common leafminer in experimental plantings of lettuce, but that *L. trifolii* predominated in commercial lettuce fields that were subject to intensive insecticide applications.

Gao et al. [7] specifically compared the differential effects of avermectin on *L. sativae* and *L. trifolii*, using populations collected from Hainan Province. They reported that *L. trifolii* was significantly more tolerant to avermectin than was *L. sativae*. Other comparative studies have generally shown that *L. trifolii* is less susceptible to insecticides than is *L. sativae* [14]–[16]. An exception to these general trends is a study conducted with Japanese populations of these species that found either no significant difference in susceptibilities, or lower susceptibilities of *L. sativae*, to 25 different insecticides [17].

Japan is one region where *L. sativae* has successfully displaced *L. trifolii*. This different displacement pattern has been attributed to the greater fecundity of *L. sativae* compared with *L. trifolii* and a lower susceptibility to a common parasitoid, in addition to the differences in insecticide susceptibility [3], [18].

Multiple mechanisms are likely to contribute to the displacement of one species by another, and it is difficult to separate effects of different mechanisms in field experiments. Therefore, we do not preclude differential parasitism as a contributing factor. It has been well documented that overuse of insecticides induces outbreaks of *Liriomyza* spp. by eliminating parasitoid populations that can suppress *Liriomyza* [19]–[21]. In general, avermectin has proven to be toxic to parasitoids of *Liriomyza* spp., but impacts of its use on leafminer pest management have been equivocal [22], [23]. However, differential parasitism would explain the increased predominance only if *L. trifolii* were more susceptible to parasitization than *L. sativae*. Surveys show that *L. sativae* has a rich and diverse parasitoid community in China [24] and other studies have shown that *L. sativae* may be less susceptible to some common parasitoids than is *L. trifolii* [18], [25].

Regardless of the specific underlying mechanism(s), insecticide use can lead to significant changes in the *Liriomyza* species complex in China. Our results show that these changes can be influenced by the general cropping system. *Liriomyza sativae* is more susceptible to displacement in intensively managed cowpea, especially where it is grown as a monoculture. It appears to be more persistent in more complex systems with hosts where it has reproductive advantages over *L. trifolii*. For the situation in southern China, maintaining untreated hosts that are better suited for *L. sativae* may increase the likelihood of its persistence in the presence of *L. trifolii*. Given the generally greater pest problems experienced with *L. trifolii* compared with *L. sativae*, this may aid in overall leafminer pest management.

## Materials and Methods

### Subheading Ethics Statement

No specific permissions were required for these locations/activities.

The location is not privately-owned or protected in any way.

The field studies did not involve endangered or protected species.

### Surveys of *Liriomyza* spp. in commercial cowpea fields

In southern China, cowpea is mainly planted in Hainan Province, and the further away from the Hainan Province, the less cowpea is planted. Therefore, we surveyed *Liriomyza* spp. in two regions that differ in the abundance of cowpea. Hainan Province was the region with high cowpea abundance and Guangdong Province was the region with relatively low cowpea crop abundance. We conducted field surveys during 2011. Because of inherent differences in the cropping seasons, we surveyed Hainan Province from January to March, and we surveyed Guangdong Province from April to June. We surveyed eight locations (Sanya, Dongfang, Haikou, Leidong, Lingshui, Wuzhisan, Qionghai, and Danzhou Cities) across Hainan Province (Fig.1), and we surveyed eight locations (Foshan, Jiangmen, Zhongshan, Guangzhou, Shenzhen, Zengcheng, Huadu, and Huizhou Cities) across Guangdong Province (Fig.3).

Cowpea foliage containing mature larvae and/or puparia of *Liriomyza* spp. was collected at each location. Leaves were excised from plants and placed on plastic containers (40×40×20 cm) to collect puparia. Pupae were held in glass scintillation vials for adult emergence. Adults were counted and identified to species.

On each sample date at each location, we collected foliage with >30 individuals per sample from each of at least three cowpea plots that were 100–500 meters away from each other. Foliage was held in environmental chamber at 26°C,16∶8 (L:D) photoperiod where the larvae and pupae were reared to adulthood. Adults were counted and identified to species. To confirm species identifications, representative adult males were identified based on the morphology of genitalia.

### Insecticide and cropping system effects on *Liriomyza* populations

Field experiments were conducted at two sites in Siling County, located in Guangzhou City, Guangdong Province in 2011 and 2012, where *L. sativae* and *L. trifolii* were known to co-occur at the time of the experiments. One site was selected for planting cowpea only, with no other crops planted within 1 km of the site. This site was also surrounded by many tall apartment buildings. This setting is similar to the intensively-planted cowpea agroecosystems on Hainan Island. A second site in Siling County was selected for planting cowpea with towel gourd planted near the cowpea field. This arrangement is common in most regions of Guangdong Province. In early of February of both years, these same two sites in Siling County were selected for planting cowpea, where *L. trifolii* and *L. sativae* were observed occurring simultaneously.

The cowpea experiments at both field sites were arranged as randomized complete blocks with three replication of each of the two treatments, the avermectin treatment and the control. Each plot had four rows with approximately 0.002 ha and seeded at a rate expected to produce 140 plants per plot (70,000 per hectare), as is common in this farming system. A 2 m space was set up between plots to minimize insecticide contamination among treatment plots. Cowpeas were maintained using the standard agronomic practices of the local areas, and no other insecticides were used throughout the season. The insecticide treatment was avermectin applied at 4.5 ppm [A.I.], and the control consisted of distilled water. A hand-pump type sprayer used to apply the treatments. It was calibrated to deliver 1300 L/ha at 20–30 kPa, with 90 μm openings in the nozzles.

For towel gourd, three plots were planted near the cowpea crops at the second site in Siling County. Each towel gourd plot contained five rows of plants, with a total area of approximately 0.01 ha. Plots were seeded at a rate to produce 150 plants per plot (15,000 plants per hectare), as is common in this type of farming system. Towel gourd was maintained using the standard agronomic practices of the local areas, and no insecticides were used throughout the season.

For the cowpea crop at both sites, sampling for leafminers and insecticide applications began in early April, approximately 8 wk after sowing. Sampling for leafminers was conducted on days 1, 7, 13, and 19 of the experiments. Insecticide applications were made after leafminer samples were collected on days 1, 7 and 13 of the experiment. Therefore, samples were collected before insecticide treatments began, and then every six days after each of the three applications. Five sites were randomly chosen within each plot on each sampling date, with a total of 50–200 leaves containing late-instar larvae collected per plot per date. Leaves were excised and placed in plastic containers (40×40×20 cm) to collect puparia. Pupae were held in glass scintillation vials for adult emergence. Adults were counted and identified to species.

After samples were collected on day 19, very few *L. sativae* were found in the insecticide treated cowpeas compared with the control treatments. To determine if leafminer populations changed after avermectin was no longer applied, we removed all of the cowpea plants in the control plots at both sites, and kept the cowpea plants in the insecticide treated plots. Thirty days later, we sampled for leafminers at both sites in the manner described above. This sampling enabled us to determine how leafminer populations changed following the removal of insecticide pressure and if the residual populations of *L. sativae* would reestablish.

For towel gourd, sampling for leafminers began in early April, approximately 8 wk after sowing. Sampling was conducted on days 1, 7, 13, 19, 25, 31, 37, 43, and 49 of the experiments. Five sites were randomly chosen within each plot on each sampling date, with a total of 25 leaves (5 leaves/site) containing late-instar larvae randomly collected per plot per date. Leaves were excised and placed in plastic containers (40×40×20 cm) to collect puparia. Puparia were held in glass scintillation vials for adult emergence. Adults were counted and identified to species.

### Statistical analyses

We used χ^2^tests to determine if there were biases in proportions of *Liriomyza* species in the field surveys of Guangdong and Hainan provinces. We used logistic regression analyses to determine if the proportion of *L. trifolii* in cowpea changed over time in association with insecticide application treatments in the different cropping systems. Because each experiment was conducted in a unique environment, data for each experiment were analyzed separately. To determine if there was an insecticide effect, we used a model with the two levels of insecticide (avermectin-treated and untreated), sample day (day 1, day 7, day 13, day 19) and the insecticide x day interaction. We used a set of linear trend contrasts to determine if the proportion of *L. trifolii* in each insecticide treatment changed in a linear manner with time. We also used contrasts to determine if the mean proportion of *L. trifolii* differed between insecticide treatments on specific sample dates. To determine if the proportions of *L. trifolii* changed after insecticide applications were halted, we compared proportions of *L. trifolii* from the avermectin treated plots on day 19 and day 49 (36 days after the final insecticide application). We also used a linear trend analysis to determine if the proportion of *L. trifolii* changed over time in the towel gourd plots. All logistic analyses were conducted using the Proc Glimmix procedure (SAS version 9.4, SAS Institute, 2012).
